# Bridge-layered decompression technique for vertebral artery-involved hemifacial spasm: technical note

**DOI:** 10.1186/s12893-024-02415-1

**Published:** 2024-05-14

**Authors:** Lei Shen, Jingyi Yang, Runqi Cheng, Chuqiao Yue, Tiansheng Wang, Songshan Chai, Yuankun Cai, Yixuan Zhou, Dongyuan Xu, Yu Lei, Mengyang Wang, Zhimin Mei, Jingwei Zhao, Xuan Dai, Bangkun Yang, Jincao Chen, Yanbing Yu, Nanxiang Xiong

**Affiliations:** 1 Department of Neurosurgery, Zhongnan Hospital of Wuhan University, No.169, Donghu Road, Wuhan, 430071 Hubei China; 2 Department of Neurosurgery, Sino-Japanese Friendship Hospital, Beijing, 100029 China

**Keywords:** Decompression, Hemifacial spasm, Microvascular decompression, Transposition, Vertebral artery

## Abstract

**Background:**

Hemifacial spasm (HFS) is most effectively treated with microvascular decompression (MVD). However, there are certain challenges in performing MVD for HFS when the vertebral artery (VA) is involved in compressing the facial nerve (VA-involved). This study aimed to introduce a “bridge-layered” decompression technique for treating patients with VA-involved HFS and to evaluate its efficacy and safety to treat patients with HFS.

**Methods:**

A single-center retrospective analysis was conducted on the clinical data of 62 patients with VA-involved HFS. The tortuous trunk of VA was lifted by a multi-point “bridge” decompression technique to avoid excessive traction of the cerebellum and reduce the risk of damage to the facial-acoustic nerve complex. To fully decompress all the responsible vessels, the branch vessels of VA were then isolated using the “layered” decompression technique.

**Results:**

Among the 62 patients, 59 patients were cured immediately after the surgery, two patients were delayed cured after two months, and one had occasional facial muscle twitching after the surgery. Patients were followed up for an average of 19.5 months. The long-term follow-up results showed that all patients had no recurrence of HFS during the follow-up period, and no patients developed hearing loss, facial paralysis, or other permanent neurological damage complications. Only two patients developed tinnitus after the surgery.

**Conclusion:**

The “bridge-layered” decompression technique could effectively treat VA-involved HFS with satisfactory safety and a low risk of hearing loss. The technique could be used as a reference for decompression surgery for VA-involved HFS.

## Introduction

Hemifacial spasm (HFS) is characterized by recurrent, paroxysmal, involuntary twitching of the unilateral facial muscles innervated by the facial nerve [1, 2]. It is believed that vascular compression of the facial nerve’s root entry zone (REZ) is the cause of HFS [3, 4]. Microvascular decompression (MVD) is the most effective treatment for patients with HFS, as it decompresses the responsible vessels [5–7]. Studies have shown that the anterior inferior cerebellar artery, posterior inferior cerebellar artery, and vertebral artery (VA), of which 10–20% are VA and its branch vessels, are the primary responsible vessels for compressing the REZ of the facial nerve [8].

However, MVD is less effective for HFS when the vertebral artery (VA) is involved in compressing the facial nerve (VA-involved) [9, 10], possibly due to the thick or tortuous trunk of the VA in patients with VA-involved HFS, making it difficult to push away from the REZ of the facial nerve [11, 12]. Additionally, the VA and its branch vessels may be the responsible vessels for the compression on the REZ of the facial nerve at the same time. If the branch vessels are missed during surgery, the decompression would be incomplete [13]. Excessive traction of the cerebellum to expose the REZ increases the risk of damaging the facial-acoustic nerve complex, which can cause facial paralysis, hearing loss, and other complications [10, 14]. Therefore, MVD for patients with VA-involved HFS is challenging, with a low cure rate and a high incidence of postoperative complications [15]. Consequently, it is necessary to develop more effective and safer decompression techniques.

Here, we introduce our center’s clinical practice of a “bridge-layered” decompression technique for treating patients with VA-involved HFS and evaluate the safety and efficacy of this technique. The report is as follows.

## Methods and materials

### Patient selection

The clinical data of patients with VA-involved HFS who underwent the “bridge-layered” decompression technique at Zhongnan Hospital of Wuhan University from September 2020 to January 2022 were retrospectively analyzed. The inclusion criteria for this study were as follows: (1) the patient was diagnosed with primary HFS based on clinical symptoms and MRI. (2) VA was confirmed as the responsible vessel for compression of the facial nerve by both preoperative MRI and intraoperative findings.

The preoperative diagnosis criteria for HFS included: (1) characteristic clinical symptoms such as recurrent paroxysmal involuntary twitching of one or both facial muscles, including orbicularis oculi muscle, muscles of facial expression, and orbicularis oris muscle, that was exacerbated by emotional agitation or tension. (2) CT and MRI ruled out possible secondary HFS caused by intracranial lesions. (3) other diseases that may cause facial muscle cramps, including Meige syndrome, idiopathic blepharospasm, and Tourette’s syndrome, were excluded by clinical signs and symptoms.

A total of 62 patients with VA-involved HFS were included in this study, with 41 cases on the left side and 21 on the right side. Among them, 35 patients were male, and 27 were female. The mean age of the patients was 53 years, ranging from 24 to 67 years old. The mean duration of spasms in patients was 3.4 years, ranging from 0.8 to 7.3 years. Before surgery, no abnormal hearing was reported in all patients.

### Preoperative evaluation

**Fig. 1:**
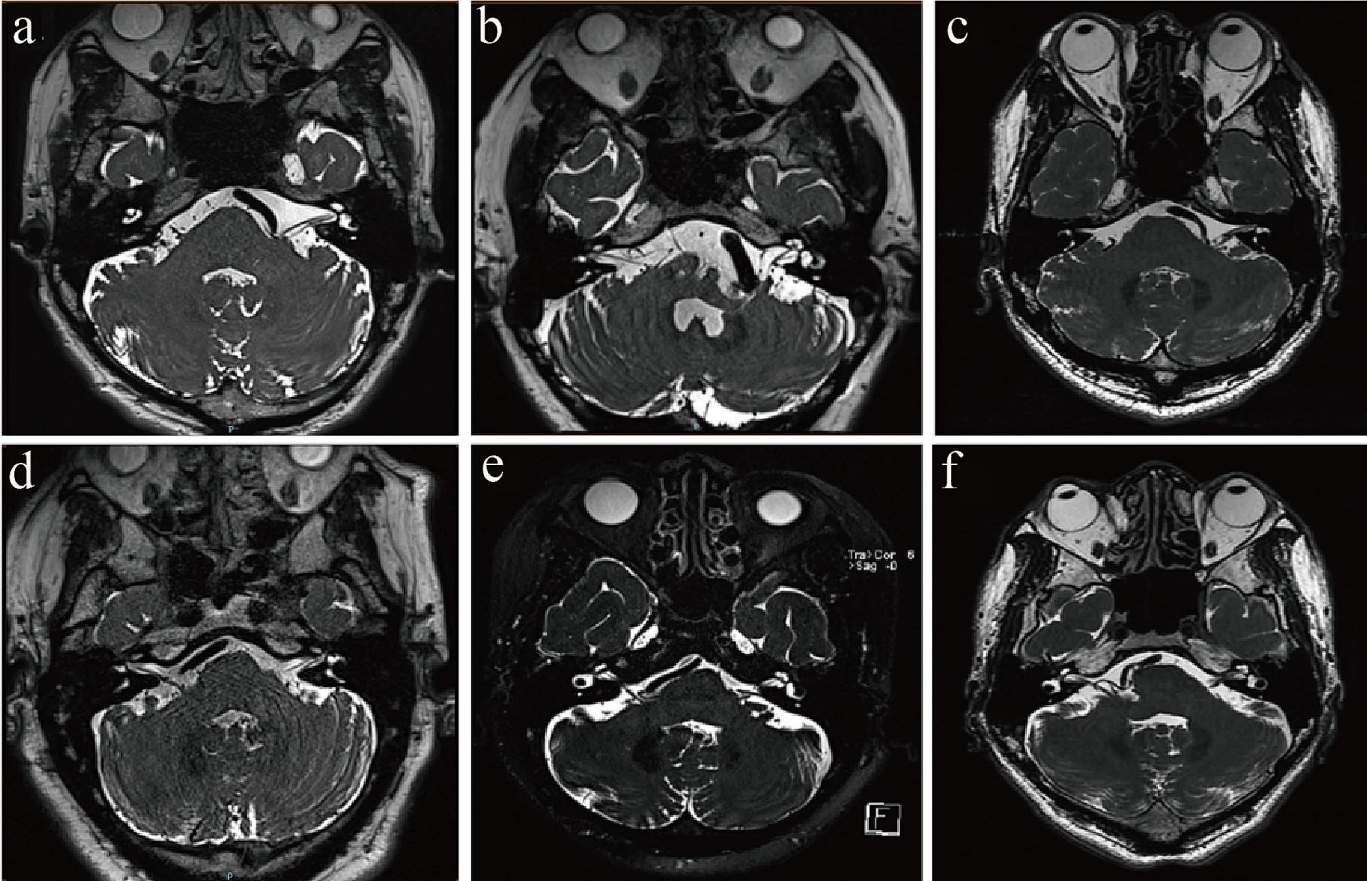
Preoperative MRI images of VA-involved patients. The images demonstrated that the REZ of the facial nerve was compressed by the VA. Panels **a**-**c** show left-sided HFS, while panels **d**-**f** depict right-sided HFS

Preoperative MRI (including MRI T1WI, T2WI, and 3D-TOF MRA) was used to confirm the VA as the responsible vessel for compression of the REZ of the facial nerve in patients with HFS (Fig. 1).

### Surgical technique

**Fig. 2:**
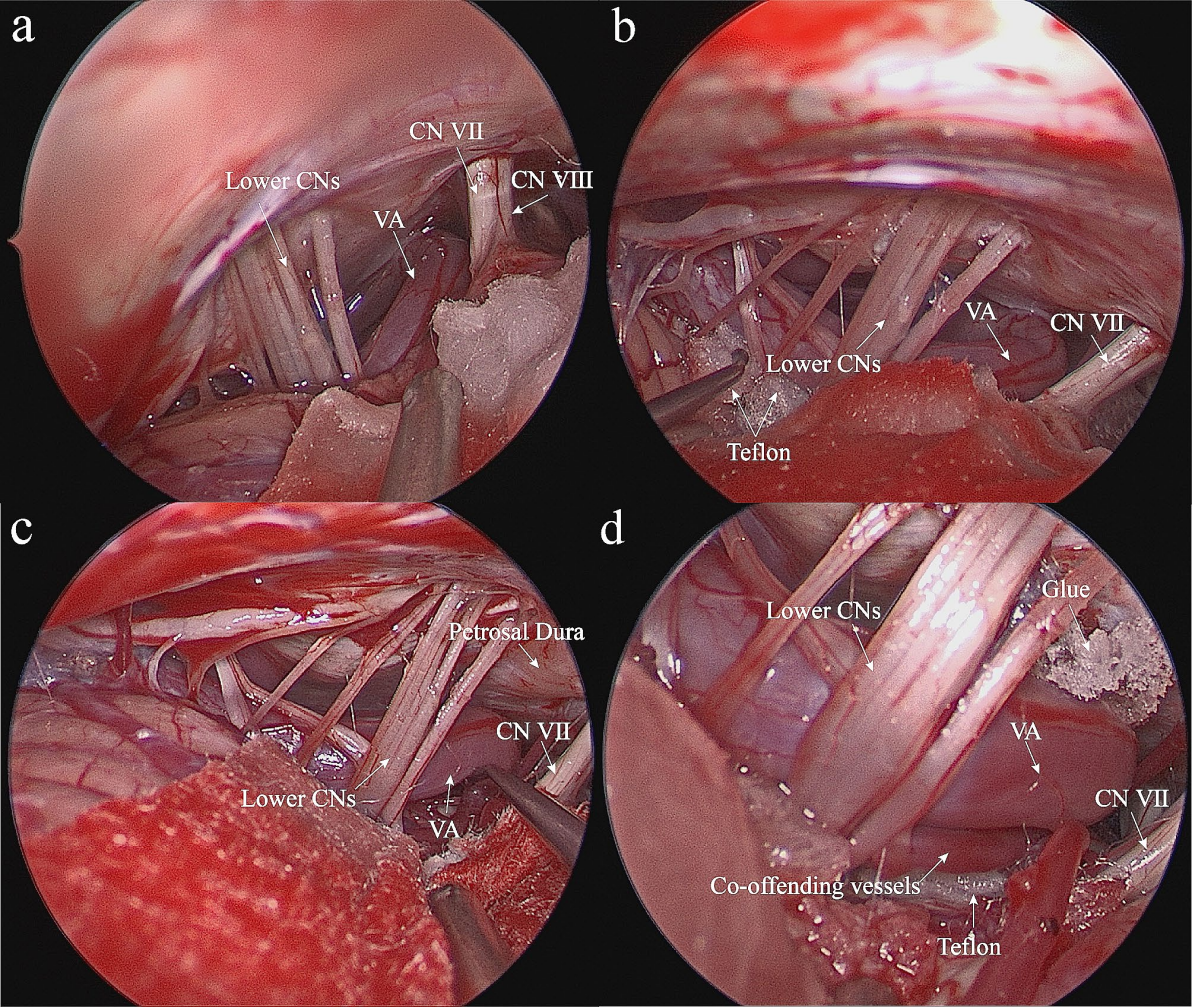
Bridge-layered decompression technique surgical process. Panel **a** displays the exposed operative field following the retrosigmoid approach. Panel **b** illustrates lifting the proximal VA by Telfon cottons. In panel **c**, the distal VA is suspended using medical bioprotein glue, fully exposing the REZ of the facial nerve. Panel **d** shows the “layered” decompression technique, relieving the facial nerve from the compression by the branch vessels of VA, ultimately leading to decompressing REZ completely. Key anatomical structures include VA (vertebral artery), CN VII (facial nerve), CN VIII (auditory nerve), and lower CNs (lower cranial nerves)

The “bridge-layered” decompression technique for VA-involved HFS was performed using the retrosigmoid approach. After a small incision craniotomy and release of the cerebrospinal fluid to collapse the cerebellum, the technique proceeded in three steps:

1) building proximal “bridge”: the arachnoid surrounding the lower cranial nerves to the facial-acoustic nerve complex and surrounding blood vessels was released (Fig. 2-a). The course of the lower cranial nerves and the VA was explored, and a Teflon cotton was placed between VA and the medulla oblongata through the gap between the lower cranial nerves. Using several Teflon cottons as piers, the VA was lifted like a “bridge” (Fig. 2-b). The goal of this step was to support and lift the proximal end of the VA, reducing the traction of the cerebellum and the facial-acoustic nerve complex, and protecting the facial-acoustic nerve complex.

2) building distal “bridge”: after building the proximal bridge, the relationship between the distal end of the VA and the REZ of the facial nerve was observed. If the VA was still very close to the REZ of the facial nerve, the step “building distal ‘bridge’” was performed. Medical bioprotein glue (Guangzhou Baiyun Medical Adhesive Co.) was used to anchor the distal end of the VA to the dura of the petrosal temporal bone between the glossopharyngeal nerve and facial nerve, sufficiently distancing the VA from the REZ of the facial nerve (Fig. 2-c). At this point, the trunk of the VA constituted the main body of the “bridge”, and the Teflon cottons, and the medical bioprotein glue constituted the support structure of the “bridge,” together forming a bridge.

3) local “layered”: after building the “bridge”, the VA was lifted away from the brainstem, exposing a surgical field that confirmed the compression of the REZ of the facial nerve by the below branch vessels of the VA. Several Teflon cottons were placed between the medulla oblongata and these responsible vessels to adequately decompress the REZ of the facial nerve (Fig. 2-d).

Intraoperative neurophysiological monitoring techniques, including brainstem auditory evoked potentials and lateral spread response (LSR), were used to monitor neural function and abnormal muscle response. The criteria for ending the surgery: (1) all responsible vessels have been decompressed. (2) the LSR completely disappeared.

### Postoperative evaluation

The surgical outcomes of the patients were evaluated based on the Cohen grade, as presented in Table 1, using clinical symptoms as the basis.

All patients received postoperative examinations to assess for complications, including CT scans to evaluate for intracranial hematoma and pneumatosis, as well as clinical symptoms and physical examination to detect cerebrospinal fluid leakages, intracranial infection, and neurological dysfunction, such as facial nerve palsy and posterior cranial nerve dysfunction. Patients who experienced hearing loss or tinnitus underwent pure tone audiometry, speech audiometry, and impedance audiometry to evaluate the degree of hearing impairment. Recovery of symptoms was assessed after twelve months to identify any permanent neurological damage.

**Table 1:** Cohen grade for clinical grading of patients with hemifacial spasm

Grade	Symptom
Grade 0	No spasms
Grade I	Increased blinking or mild tremor of facial muscles caused by external stimuli
Grade II	Eyelid, facial muscle twitching spontaneously, no dysfunction
Grade III	Severe spasms and mild dysfunction
Grade IV	Severe spasms and dysfunction (inability to drive, read, etc.)

### Follow-up

Follow-up methods included both outpatient and telephone follow-up, with a minimum follow-up period of twelve months. During the follow-up period, patients underwent routine gross sound audiometry and facial nerve function tests. Patients with hearing loss or tinnitus also underwent additional audiometry tests, while facial nerve conduction was assessed using electromyography, and secondary facial palsy was ruled out using MRI.

### Statistical analysis

Statistical analyses were performed using GraphPad Prism (version 9.0.0). The Chi-square test was used to compare the differences between our results and the results of the existing data. Two-sided *p* ≤ 0.05 (*) was considered statistically significant.

## Results

### Short-term results

95% of patients (59/62) were immediately healed after surgery, with complete disappearance of HFS symptoms and no recurrence. Delayed cure was observed in 3% of patients (2/62). Additionally, 2% of patients (1/62) experienced occasional spontaneous facial muscle twitching after surgery, with a Cohen grade of II.

The postoperative evaluation showed that 60 patients were in good condition with no postoperative complications, such as intracranial hematoma, infection, or neurological dysfunction.

### Long-term results

Patients were followed up for an average of 30.0 months, ranging from 22 to 38 months, during which no HFS recurrence was observed in any patients. The delayed cure was observed in two patients, with significant symptom relief after surgery and complete resolution of HFS symptoms within two months. Two patients developed tinnitus, one week and one month after surgery, respectively. Among them, one patient experienced significant symptom relief after taking mecobalamin, while the other patient’s tinnitus disappeared after taking mecobalamin. Facial pain/headache occurred in three patients after surgery, which resolved within three months. No facial paralysis or hearing loss was reported, and none of the patients had permanent neurological damage complications.

To compare the differences between our results and the results of the existing data, we systematically collected the cohort studies of VA-involved HFS published from 2008 to 2022. The results of these cohort studies are summarized in Table 2. Based on these results, the mean surgical cure rate for VA-involved HFS was 90.1%, the mean long-term postoperative complication incidence was 7.1%, and the mean recurrence rate was 2.8%. In our study, the cure rate was 98.4%, which was higher than the mean cure rate of previous studies (*p* = 0.02), while long-term postoperative complication incidence (0.0%) (*p* = 0.03) and recurrence rate (0.0%) (*p* = 0.40) were lower than previous studies. These results indicate that the bridge-layered technique is superior to the average of previously reported studies in terms of effectiveness and safety.

**Table 2:** The results of previously reported cohort studies of vertebral artery-involved hemifacial spasm

Studies	Years	Technique	Number of patients	Cure rate/%	Complication incidence#/%	Recurrence rate/%
Dai et al. [16]	2015	MVD	133	-	-	-
Mikami et al. [17]	2013	MVD	18	94.4	-	-
Xue et al. [18]	2021	MBG^ anchor	33	100	13.0	-
		MVD	54	85.2	18.0	-
Jiang et al. [19]	2020	MVD	198	97.5	17.7	1.0
Zhang et al. [20]	2017	MVD	153	86.7	0.7	-
		MBG anchor	174	95.8	2.4	-
Ferreira et al. [21]	2011	Sling anchor	6	100.0	16.7	-
Lee et al. [22]	2016	MBG anchor	42	100.0	2.4	0.0
Zaidi et al. [23]	2015	MVD	17	82.4	5.9	11.8
Kim et al. [24]	2008	MVD	79	86.1	1.3	3.8
Kim et al. [25]	2019	PTTI	16	81.3	0.0	-
		MVD	6	50.0	33.3	-
Masuoka et al. [26]	2017	MVD	22	86.4	22.7	-
Jiang et al. [27]	2018	MBG anchor	117	94.9	-	-
Kim et al. [15]	2012	MVD	232	89.2	4.4	1.3
Yang et al. [28]	2017	MVD	29	89.7	13.8	-
Shimano et al. [29]	2015	MVD	33	51.5	6.1	0.0
Nonaka et al. [10]	2019	Wedge	13	92.3	-	7.8
Inoue et al. [9]	2021	Bridge	30	96.7	10.0	3.3
		MBG anchor	30	83.3	23.3	0.0
Wang et al. [13]	2022	MVD	109	87.2	7.3	9.2
Lee et al. [14]	2021	MVD	271	88.9	3.7	-
Average*	-			90.1	7.1	2.8
Our study	2023	Bridge-layered	62	98.4	0.0	0.0
			*P* value	0.02	0.03	0.4

#. Complication incidence: long-term postoperative complication incidence

^. MBG: Medical bioprotein glue anchor

*. Average: Average results of previous studies

## Discussion

For VA-involved HFS, decompression techniques reported in the past can be classified as isolation and transposition techniques [25, 30–32]. The isolation technique involves placing an implant between the REZ of the facial nerve and the responsible vessel to relieve compression, which is easier to perform. However, previous studies have found that the disadvantage of the isolation technique is insufficient decompression, which may lead to recurrence [11]. Therefore, the transposition technique is currently the preferred treatment for VA-involved HFS. For VA-involved HFS, the transposition technique involved anchoring the VA in a new position, leaving enough space between the facial nerve and the responsible vessel, interrupting the transmission of pulse force, and freeing the facial nerve from neurovascular conflict. Theoretically, the transposition technique can avoid the formation of adhesions and granulomas around the decompression site, and reduce the risk of recurrence of HFS after surgery by shifting the VA away from the REZ of the facial nerve.

However, many previously reported transposition techniques are complicated, time-consuming, and challenging to master. For example, the Wedge-technique achieves VA transposition by inserting one or more Teflon cottons folded into a rod or wedge shape between the VA and the brainstem or cerebellar hemispheres and using fibrin glue to firmly adhere the VA to the Teflon cottons [10]. Alternatively, the aneurysm clip suspension method uses a vascular sling and a non-absorbable dural tape for VA transposition, followed by fixation of the VA suspension complex to the dura on the rock bone by means of an aneurysm clip across the dural bridge [33]. Additionally, the silk suspension method uses a “neck tie” technique to remove the VA from the facial nerve and then sutures the sling to the dura [34]. These transposition techniques are either challenging to master, resulting in longer operation times, or leave non-absorbable foreign bodies, leading to rejection reactions.

Additionally, some transposition techniques have a higher incidence of complications such as hearing loss and facial palsy [20]. This is mainly due to the higher risk of damaging the facial-acoustic nerve complex caused by excessive traction of the cerebellum to expose the REZ of the facial nerve [10, 14]. To reduce the risk of damaging the facial-acoustic nerve complex, the current technique proposes operating first in the gap between the lower cranial nerves and lifting the proximal VA, which also enables lifting the distal VA that compresses the REZ of the facial nerve. This allows for easier exposure of the REZ of the facial nerve, without the need to excessively tract the facial-acoustic nerve complex. Long-term follow-up results show that no patients experienced postoperative hearing loss or facial paralysis, indicating that the “bridge-layered” decompression technique has a lower risk of damaging the facial-acoustic nerve complex.

It should be noted that the responsible vessels of patients with VA-involved HFS usually include the tortuous VA and its branch vessels. If only the trunk of the VA is decompressed using the “bridge” technique, its branch vessels may continue to compress the REZ of the facial nerve, resulting in poor surgical efficacy or postoperative recurrence [13]. Therefore, after fully exposing the surgical field, it is necessary to decompress the branch vessels of the VA layer by layer, ensuring that all responsible vessels are fully decompressed. In the current study, 98% of the patients’ symptoms were effectively controlled, and the other 2% had occasional facial muscle twitching after the surgery, which means that the “bridge-layered” decompression technique could effectively treat patients with VA-involved HFS.

Compared to previously reported transposition techniques for treating VA-involved HFS, the “bridge-layered” decompression technique is easier to operate. Firstly, this technique operates through the gap between the lower cranial nerves without excessively tracting the cerebellum. What’s more, lifting the proximal VA by the “bridge” technique makes it easier to lift the distal VA that compresses the REZ of the facial nerve. Finally, when the gap between the REZ is limited, the VA can be suspended by medical bioprotein glue, which not only effectively anchors the VA but also allows more space for “layered” decompression of the branch vessels of the VA.

Attention should be paid to the following points when performing the “bridge-layered” decompression technique: (1) the VA has greater stiffness and a thicker diameter, which can obstruct the surgical field and make it difficult to move. Therefore, the surgeon should move the VA gently through the gap between the lower cranial nerves to avoid excessive traction of the cerebellum, which could lead to postoperative complications. (2) The use of biological glue for transposing the VA must be careful. The amount of biological glue instilled should be appropriate, with no overflow or dripping, to avoid chemical vasculitis and damage to peripheral nerves.

However, this study has some limitations. First, it was a retrospective study subject to inherent biases in the study design. Second, this was a single-center study, and the surgeries were performed by a single surgeon, indicating that the surgical effect may be affected by the surgeon’s proficiency. Furthermore, the number of cases and observation indicators included in this study were small, and there was no control group. Therefore, larger-scale prospective studies are needed to further explore the safety and effectiveness of the “bridge-layered” decompression technique.

## Conclusion

This study presents a novel “bridge-layered” decompression technique for treating VA-involved HFS. The “bridge” technique frees the REZ of the facial nerve from the compression of the thick and tortuous VA trunk. Subsequently, the “layered” decompression technique is utilized to completely relieve the compression of the branch vessels, resulting in full decompression of all the responsible vessels. The study demonstrated that the “bridge-layered” decompression technique is an effective treatment for VA-involved HFS and has the potential for wider clinical application.

## Data Availability

The clinical data of patients in the current study were extracted from electronic health records in the medical record system of Zhongnan Hospital of Wuhan University. The clinical data could be obtained through Dr. Shen at Zhongnan Hospital of Wuhan University.
